# The rehydration behavior of microwave‐dried amaranth (*Amaranthus dubius*) leaves

**DOI:** 10.1002/fsn3.406

**Published:** 2016-07-07

**Authors:** Saheeda Mujaffar, Alex Lee Loy

**Affiliations:** ^1^ Food Science and Technology UnitDepartment of Chemical EngineeringThe University of the West IndiesSt. AugustineTrinidad and Tobago

**Keywords:** Amaranth, microwave drying, Peleg's equation, rehydration

## Abstract

The effect of temperature (35, 50, and 60°C) on the rehydration behavior of microwave‐dried amaranth (*Amaranthus dubius*) leaves was investigated. Leaves were dried at 700 W power level before rehydrating in water. The higher the rehydration temperature, the higher the equilibrium moisture content of the leaves, although the effect was not statistically significant. The increase in rehydration ratio was significant only as temperature increased from 50 to 60°C. The process was adequately described by the Peleg sorption model, with the Peleg rate constant (*K*_1_) and the Peleg capacity constant (*K*_2_), both decreasing as rehydration temperature increased. While the color difference (*ΔE*) between fresh leaves and leaves rehydrated at 35°C was significantly higher than for the leaves rehydrated at 50 and 60°C, this difference was not visible. Cooking of leaves occurred beyond 120 min at the higher rehydration temperatures . Based on the results, rehydration of microwave‐dried leaves was successfully carried out at 35°C, however, rehydrated leaves were darker than the fresh leaves. Increasing the temperature to 50°C improved the rehydration capacity and the color of the leaves, however, cooking of leaves occurred by the second hour of the process.

## 1 Introduction

The genus Amaranth consists of approximately 60 plant species, some of which are used as food grain, leafy vegetables, and ornamentals (Borneo & Aguirre, 2008). Vegetable amaranth (*Amaranthus* sp.) grows easily in the Caribbean and is widely used throughout the region interchangeably with spinach (*Spinacia oleracea* L) as a leafy green. The species commonly found in Trinidad and Tobago is *Amaranthus dubius* (Mohoyodeen, 1995). As is typical of leafy greens, the postharvest life of amaranth is relatively short because of rapid wilting under tropical ambient conditions. Interest in extending the shelf life and adding value to this perishable leafy green led to research work on the microwave drying, and subsequent rehydration of amaranth leaves. Previous research works on dried amaranth have focused primarily on quality and nutrient content of the dried material (Aletor & Abiodun, 2013; Fathima, Begum, & Rajalakshmi, 2001; Peter, Elizabeth, Judith, & Hudson, 2014; Rodriguez, Perez, Romel, & Dufour, 2011) and Borneo and Aguirre (2008) looked at the use of the ground, dried amaranth leaves in green pasta as a spinach substitute. Akonor and Amankwah (2012) and Singh, Singh, Singh, Singh, and Singh (2014) looked at various aspects of drying kinetics of sun and solar‐dried amaranth and Fathima et al. (2001), Rajeswari, Bharati, Ramchandranaik, and Naganur (2013) and Pati, Pardeshi, and Shinde (2015) investigated the microwave‐drying potential of amaranth leaves specifically.

Microwave drying of food materials is becoming an increasingly popular alternative to conventional hot air drying techniques due to the advantages such as a reduction in drying time, lower impact on product quality, and less energy use (Zhang, Tang, Mujumdar, & Wang, 2006). Food dehydration using microwaves specifically targets the water molecules present in the material and these molecules absorb energy which is converted to heat. An internal pressure gradient therefore results and water is “pumped” to the material surface from where it evaporates, resulting in drying of the material (Decareau, 1985; Maskan, 2001; Zhang et al., 2006). Since the transfer of energy during microwave processing does not rely on diffusion of heat from the surfaces as during conventional heating, it is possible to achieve rapid and uniform heating (Venkatesh & Raghavan, 2004).

Dehydrated foods are generally rehydrated prior to or undergo rehydration during use. With respect to dried amaranth leaves, not much has been reported in the literature on rehydration. Fathima et al. (2001) investigated the rehydration of various microwave‐dried greens, including amaranth (*Amaranth* sp.). Leaves were first blanched in hot water at 98°C for 2 min, then microwave dried at 100% power for 12 min. Rehydration involved soaking the dried greens in distilled water (temperature not given) sufficient to cover the material. For amaranth leaves, they reported a reconstitution time of 60 min and a reconstitution ratio, calculated as the change in weight of the reconstituted greens as a percentage of the weight of the fresh greens of 38.3%. Rajeswari et al. (2013) compared the rehydration ratio of microwave‐dried and air‐dried amaranth leaves. They found that the samples dried in a cabinet drier without any pretreatment exhibited a higher rehydration ratio of 5.60, while microwave‐dried samples had a lower rehydration ratio of 4.73. Rehydration temperature was not given. Pretreatments such as blanching and sodium bisulfite prior to drying generally resulted in leaves having lower rehydration ratios.

With respect to the rehydration kinetics following microwave drying of other leafy materials, Dadali, Demirhan, and Ozbek (2008) investigated the effect of microwave drying conditions on the rehydration kinetics of spinach leaves at different temperatures (30–70°C) for 5 hr. They fitted the rehydration data to two models, the Peleg model and the Weibull model, finding that the Peleg model gave the better fit. They reported a Peleg rate constant (*K*_1_) of 45.5605 (s per g H_2_O/g DM) and Peleg capacity constant (*K*_2_) of 0.2764 (1/g H_2_O/g DM) for spinach leaves dried at 720 W power. Demirhan and Ozbek (2010) investigated the effect of drying conditions and rehydration temperatures on rehydration kinetics of microwave‐dried basil. Of the four rehydration models tested, they found the Weibull model to best fit the rehydration data. They reported a decrease in Peleg rate constant (*K*_1_) and Peleg capacity constant (*K*_2_) as rehydration temperature increased from 30 to 70°C.

The rehydration characteristics of dried foods give an indication of the physical and chemical changes that may have occurred during the drying process (Doymaz, 2014) and there are many factors which can affect the rehydration behavior of dried materials. As pointed out by Dadali et al. (2008), the objective of a rehydration study was to obtain products with as much original textural characteristics in as short time as possible. Three events occur simultaneously during the rehydration process: the absorption of water into the dried material, swelling of the tissue, and leaching of soluble solids back into the rehydrating solution. The rehydration kinetics of dried foodstuffs is of critical importance to their sensory properties and delivery of flavor and functional molecules (Weerts, Martin, Lian, & Melrose, 2005). Given the lack of information on the rehydration characteristics of amaranth leaves, the objective of this work was to investigate the effect of rehydration temperature on the behavior of microwave‐dried amaranth (*Amaranthus dubius*) leaves.

## 2 Materials and Methods

### 2.1 Raw material handling and preparation

Amaranth shoots (*Amaranthus dubius*) purchased at the nearby wholesale market at Macoya, Trinidad, were stored at 4°C in a refrigerator until use (Dadali et al., 2008; Ozkan, Akbudak, & Akbudak, 2007). The leaves were separated from the main hardy stems and damaged or wilted leaves were removed. The weight, length, and width of leaves were averaged 2 g, 11 cm, and 6 cm, respectively.

### 2.2 Microwave drying

Drying was carried out using a 34 L oven capacity Amana MCS10TS Menumaster Commercial digital microwave oven (Accelerated Cooking Products (ACP), Cedar Rapids, IA, USA) with the following technical features: 3.5 kV, 1,000 W, 120 V, 60 Hz. Based on studies on the microwave drying of leaves at 200–1000 W (Lee Loy, 2015), an optimum microwave power level of 700 W was chosen to produce dried samples for the rehydration study. Leaves were dried in several batches of 20 g each until constant weight was achieved, which was accomplished after 780 s. At the end of drying, the leaves were allowed to cool, packaged in resealable plastic storage bags, and stored in laboratory desiccators until rehydration.

### 2.3 Rehydration

The rehydration characteristics of dried amaranth leaves were investigated at 35, 50, and 60°C using a 26.5 L capacity Precision Reciprocating Shaker Water Baths (Model 2872, Thermo‐Scientific, Ohio, USA) with a stainless steel chamber and temperature range of ambient to 99.9°C ± 0.1°C. The operating temperature at under ambient conditions was recorded as 35°C. The selection of rehydration temperatures of 50 and 60°C was based on similar studies on the rehydration of food materials (Maharaj & Sankat, 2000; Dadali et al., 2008; Vega‐Gálvez, Notte‐Cuello, Lemus‐Mondaca, Zura, & Miranda, 2009; Balasubramanian et al., 2011; Zura‐Bravo et al., 2013) due to the complications of cooking of leaves at higher rehydration temperatures. Zura‐Bravo et al. (2013) also noted that the rehydration process in hand‐processing and commercial applications is typically carried out at temperatures below 80°C. Balasubramanian et al. (2011) found that betel leaves rehydrated at the lower temperatures of 25 and 40°C mostly resembled fresh leaves compared with leaves that were rehydrated at 80°C. Preliminary studies on the amaranth revealed that cooking of leaves and leaching of material into the rehydration solution occurred rapidly at temperatures above 60°C, therefore, 60°C was selected as the highest temperature.

When the water bath reached the required temperature, approximately 5 g of dried leaves were immersed in beakers placed in the water bath. At 5‐min intervals, the leaves were taken out from the respective beakers, drained and lightly blotted with tissue paper to remove surface water, then quickly weighed and returned to the bath. This was continued until a constant weight was recorded. The procedure was performed at each temperature in triplicates. At the end of each rehydration run, leaves were allowed to cool before packaging in resealable plastic storage bags and stored in a refrigerator at 4°C until analysis.

### 2.4 Analytical methods

Sample weights (g) during the rehydration process were recorded (0.01 ± 0.005 g) using an Explorer Pro Balance, Model EP2102C (Ohaus Corporation, NJ, USA). Moisture content of the fresh, dried, and rehydrated samples was analyzed using a Halogen Moisture Analyzer HB43‐S (Mettler Toledo‐AG, Zurich, Switzerland) at a temperature of 105°C (Dadali et al., 2008; Ozkan et al., 2007). The moisture content expressed both on a percentage of wet weight basis (g H_2_O/100 g FW) and on a dry weight basis (g H_2_O/g DM). Water activity (*a*_*w*_) was measured using an Aqua Lab CX‐2 1021 water activity meter (Decagon Devices Inco., Pullman, WA, USA). Hunter values (*L*,* a*,* b*) were recorded using a CR‐410 Chroma Meter (Konica Minolta Sensing Americas, Inc., NJ, USA). The maximum for “*L*” value is 100 (white) and the minimum is zero (black). Positive “*a*” value is red, negative “*a*” is green, while positive “*b*” value is yellow and negative “*b*” is blue (Hunter Associates Laboratory Inc., 2008). Hue angle (°), Chroma, and total color difference (ΔE) between fresh and dried leaves were calculated as given in Equation (1) through Equation (3) .

(1) $$\text{Hue}=\text{Arc}\tan\left(\frac{b}{a}\right)$$

(2) $$\text{Chroma}=\sqrt{\left(a^{2}+b^{2}\right)}$$

(3) $$\Delta E=\sqrt{{\left(L_{0}-L\right)}^{2}+{\left(a_{0}-a\right)}^{2}+{\left(b_{0}-b\right)}^{2}}$$

### 2.5 Rehydration data analysis

Weight gain at each point in time during the rehydration process was calculated as the change in weight (g) in samples divided by the original sample weight (g) prior to rehydration, expressed as percentage. Moisture content (g H_2_O/g DM) of the leaves during the rehydration process was calculated based on the dry matter content (g) of dried leaves at the start of the rehydration process, as the dry matter content is assumed to remain unchanged. The rate of change in moisture content was calculated as the change in moisture content as a function of average time (s) and expressed in g H_2_O/g DM/s. To facilitate comparison of the results of this study with those of previously reported studies, a rehydration ratio (RR) was calculated using two methods seen in rehydration studies. The first method given in Equation (4) as RR1 is commonly used as a simple indicator of the progress of the rehydration process, as the weight (g) of the rehydrated material divided by the original weight (g) of the dried material (Fathima et al., 2001; Gornicki, Kaleta, Winiczenko, Chojnacka, & Janaszek, 2013; Jokic et al., 2009; Maskan, 2001; Okpala & Ekechi, 2014). The second method given in Equation (5) is based upon the ratio between the absorbed water (g) divided by the weight (g) of dry matter in the dried material (Balasubramanian et al., 2011; Doymaz, 2014):

(4) $$\text{RR1}=\frac{{\text{Wt}}_{\mathrm{rehydrated}\;\mathrm{material},\;t=t}}{{\text{Wt}}_{\mathrm{dehydrated}\;\mathrm{material}}}$$

(5) $$\text{RR2}=\frac{\text{Wt}{\;}_{\mathrm{water}\;\mathrm{absorbed},\;t=t}}{\text{Wt}{\;}_{\mathrm{DM}}}$$

As has been done in similar works on rehydration (Maharaj & Sankat, 2000; Turhan, Sayar, & Gunasekaran, 2002; Dadali et al., 2008; Demirhan and Ozbek, 2010; Shafaei, Masoumi, & Roshan, 2014), the rehydration data in this study on amaranth leaves was modeled using the Peleg two‐parameter sorption model given in Equation (6), from which the Peleg rate constant (*K*_1_) and Peleg capacity constant (*K*_2_) was determined through a plot of the linearized form of the equation (Eq. (7)).

(6) $$M_{t}=M_{0}\pm \frac{t}{k_{1}+k_{2}t}$$

(7) $$\frac{t}{M_{t}-M_{0}}=K_{1}+K_{2}t$$

The Peleg constant (*K*_2_ *)* was then used to calculate *M*_e_ using Equation (8) (Maharaj & Sankat, 2000; Turhan et al., 2002, Demirhan and Ozbek, 2010).

(8) $$M_{e}=M_{0}+\frac{1}{K_{2}}$$

### 2.6 Statistical analysis

General analysis of variance (ANOVA) was carried out using Genstat Discovery Edition 10.3 Statistical Software (VSN International Ltd., 2011) and post hoc analysis was carried out using the software statistical software by Assaad, Zhou, Carroll, and Wu (2014): “Rapid publication‐ready MS‐Word tables for one‐way ANOVA” and “Rapid publication‐ready MS‐Word tables for two‐way ANOVA”. Drying curves were generated using Microsoft Excel 97.

## 3 Results and Discussion

### 3.1 Appearance

During the rehydration process, there were visible changes in amaranth leaf size, texture, and color. Rehydration was continued until the leaves attained constant weight, which was at a maximum of 180 min. As rehydration progressed, the leaves showed a visible increase in length and width, and the texture of the leaves changed from brittle to turgid and soft, while regaining their original shape. For all leaves, there was a color change from the light green back to the darker green color similar to the fresh leaves. After about 120 min of rehydration at 50 and 60°C, leaves developed a “cooked” aroma. At all temperatures, there was a change in the color of the rehydrating water to pale yellow, indicating that there was leaching of solids from the leaves during rehydration process. These results indicate that the leaves can be easily rehydrated in water at ambient temperature, or will also rehydrate well in food preparations that require heat. Balasubramanian et al. (2011) noted that while higher temperatures can result in higher rehydration rates, product deterioration is also increased. They found that betel leaves rehydrated at the lower temperatures of 25 and 40°C mostly resembled fresh leaves compared with leaves that were rehydrated at 80°C.

Table 1 gives the color attributes of rehydrated leaves compared with fresh and microwave‐dried leaves. The “*L”* values of microwave‐dried leaves were higher than in fresh leaves, indicating lightening of the leaves. This was also reported by Shaw, MEDA, TABIL, and OPOKU (2007) for microwave‐dried coriander leaves, who added that lightening of leaves was more pronounced in leaves dried using a convective dryer. The change in “*L*” values are an indicator for measuring browning (Dadali, Demirhan, & Ozbek, 2007) and some authors have found “*L*” values to decrease during microwave drying of parsley and spinach (Soysal, 2004; Ozkan et al., 2007; Dadali et al., 2007). The green color of amaranth leaves was intensified during microwave drying, as evidenced by a greater negative “*a*” value, and there was an increase in “*b*” value in dried leaves, indicating yellowing of the leaves. Dadali et al. (2007) reported that the changes in color attributes of microwave‐dried spinach leaves depended on the ratio of microwave output power to sample amount. Ozkan et al. (2007) reported that the least changes in color of microwave‐dried spinach were observed at a power level of 750 W as power increased from 90 to 1,000 W.

**Table 1:** Color attributes (top surface) of fresh, dried, and rehydrated amaranth leaves

Attribute	Fresh	Dried 700 W	Rehy 35°C	Rehy 50°C	Rehy 60°C
*L*	27.9 ± 0.439^a^	33.6 ± 1.07^b^	23.3 ± 0.415^d^	24.5 ± 0.568^cd^	25.2 ± 0.0994^c^
*a*	−5.85 ± 0.424^a^	−8.51 ± 0.427^b^	−1.03 ± 1.33^c^	−2.08 ± 0.0798^c^	−0.93 ± 0.806^c^
*b*	6.93 ± 0.331^a^	12.9 ± 0.631^b^	4.59 ± 0.253^c^	4.86 ± 0.382^c^	5.03 ± 0.202^c^
*Hue* (*°*)	−49.9 ± 0.74	−56.7 ± 0.0871	−31.4 ± 30.7	−66.6 ± 1.65	−71.7 ± 0.927
*ΔE*	Reference	8.77 ± 0.977^a^	7.32 ± 1.14^ab^	5.55 ± 0.603^b^	5.98 ± 1.28^b^
*Chroma*	9.07 ± 0.524^a^	15.5 ± 0.761^b^	5.22 ± 0.321^c^	5.55 ± 0.603^c^	5.3 ± 0.218^c^

Values are means ± SEM, *n* = 4 per treatment group.

Means in a row without a common superscript letter differ (*p *<* *.05) as analyzed by one‐way ANOVA and the LSD test.

It was found that *L, a*,* b,* and *Chroma* values of rehydrated leaves were significantly different (*p ≤ *.05) from the fresh leaves. The “*L*” values of all rehydrated leaves were significantly lower than the fresh leaves, as the rehydrated leaves appeared to be darker. The “*a*” values were less negative, indicating a loss of greenness and the “*b*” values were less positive, indicating a loss of yellow color. This could have been due to the leaching of color pigments into the hydrating solution, which was observed. With respect to rehydration temperature, there were no significant differences between the *Hue* or *Chroma* values of leaves. The color difference (*ΔE*) between fresh leaves and leaves rehydrated at 35°C was significantly higher than for the leaves rehydrated at 50 and 60°C. In terms of color attributes, therefore, the leaves hydrated at the higher temperatures were more favorable.

### 3.2 Moisture content

**Figure 1:**
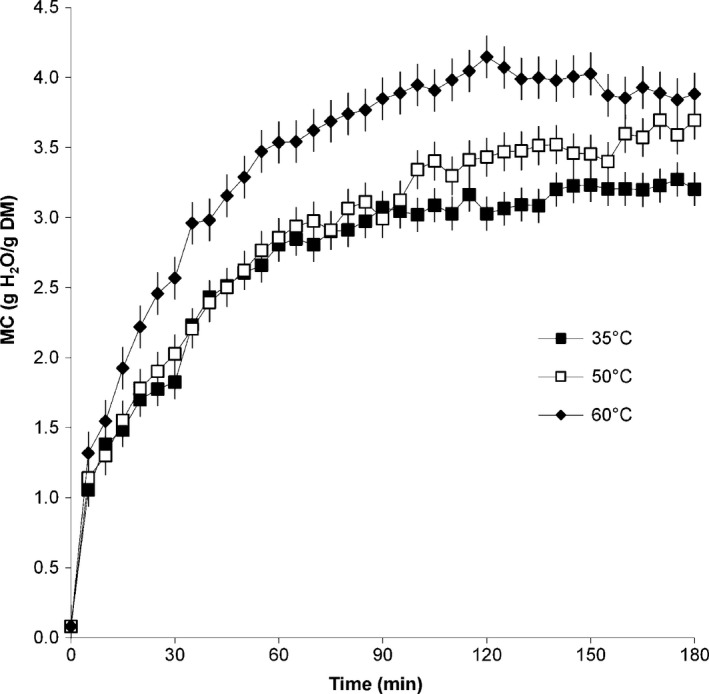
Moisture content values of amaranth leaves during rehydration at different temperatures

As expected, leaves experienced an increase in weight as rehydration progressed, with the increase being more drastic during the first 33 min then tapering off. At the end of the rehydration process, leaves that rehydrated at 60°C showed a higher final weight gain of 66% compared with leaves at 35 and 50°C with a similar average weight gain of 53%. The moisture content values of fresh and microwave‐dried leaves averaged 5.20 g H_2_O/g DM (83.9% wb) and 0.08 g H_2_O/g DM (7.6% wb), respectively. Water activity values were reduced during drying from 0.967 to 0.263. The change in moisture content with time of rehydrated leaves is given in Figure 1. Moisture content was significantly affected by time (*p ≤ *.001) and rehydration temperature (*p ≤ *.001). The equilibrium moisture and water activity values as well as the rehydration ratios at the end of the rehydration process are given in Table 2.

**Table 2:** Moisture and water activity values of fresh, dried, and rehydrated amaranth leaves

	Fresh	Dried 700 W	Rehy 35°C	Rehy 50°C	Rehy 60°C
*M* (g H_2_O/g DM)	5.20 ± 0.47^a^	0.08 ± 0.001^b^	3.20 ± 0.07^c^	3.69 ± 0.08^d^	3.88 ± 0.07^d^
*a*_*w*_	0.967 ± 0.010^a^	0.263 ± 0.019^b^	0.995 ± 0.003^a^	0.992 ± 0.004^a^	0.990 ± 0.005^a^

Values are means ± SEM, *n* = 3 per treatment group.

Means in a row without a common superscript letter differ (*p *<* *.05) as analyzed by one‐way ANOVA and the LSD test.

When the microwave‐dried amaranth leaves were placed into the water, the increase in moisture content at all temperatures was rapid in the first 5 min, after which the increase became more gradual. As expected, moisture content values followed a similar trend to the weight gain data. The moisture values of leaves at 35 and 50°C were similar until 100 min into the rehydration process, after which the moisture content values of leaves at 50°C showed a further increase, while those of leaves at 35°C tapered off. This could have possibly been due to cooking of the leaves at 50°C which resulted in increased water absorption. The moisture values for leaves at 60°C were significantly higher than for those at 35 and 50°C.

As given in Table 2, dried leaves were not rehydrated to the original moisture content of 5.20 g H_2_O/g DM. Demirhan and Ozbek (2010) attributed loss of rehydration ability in microwave‐dried basil leaves to structural changes occurring during the drying process. The higher the rehydration temperature, the higher the final moisture content of the rehydrated samples.

The moisture curves and increase in equilibrium moisture content values with increasing temperature obtained in this study are similar to those obtained by other authors, that is, with a sharp increase in moisture content at the initial phase of rehydration was followed quickly by a gradual increase until equilibrium (Maharaj & Sankat, 2000; Dadali et al., 2008; Vega‐Gálvez et al., 2009; Khazaei and Mohamadi, 2009; Demirhan and Ozbek, 2010; Shafaei et al., 2014). As also found by previous researchers, the temperature effect could not be clearly seen during the initial stages of the rehydration process (Shafaei et al., 2014; Vega‐Gálvez et al., 2009). Initial high rapid rates of water uptake were possibly due to the high moisture difference between the leaves and the medium at the start of drying. Weerts et al. (2005) used dried green tea leaf material as a model system of porous foodstuff to show the feasibility of modeling the rehydration process using the capillary flow approach. They reported that the transfer properties of water in the porous medium depend on the moisture content and the microstructure of the medium. Increase in the hydration rate with temperature was attributed mainly to the changes in viscosity and surface tension of water. Higher water absorption at higher temperatures can also be attributed to possible cellular breakdown and tissue disruption of the leaves at higher temperatures.

### 3.3 Drying rate

**Figure 2:**
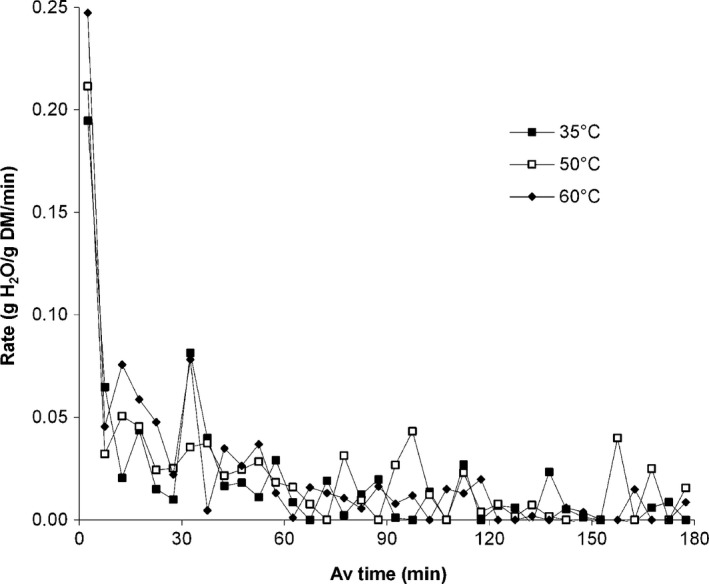
Rate of change in moisture content in amaranth leaves during rehydration at different temperatures

Drying rate curves are presented in Figure 2 reveal that the greatest impact of rehydration temperature occurred during the initial stages of the process. Initial rehydration rate averaged 0.1947, 0.2115, and 0.2474 g H_2_O/g DM/min for leaves at 35, 50, and 60°C. This was followed by a drastic drop in rate to 0.0648, 0.0321, and 0.0454 g H_2_O/g DM/min, respectively, and a very gradual decline until equilibrium was achieved. Pervin, Islam, and Islam (2008) found a similar trend in rehydration rate with respect to rehydration time for dried lablab bean seeds, with initial high rates calculated simply as the change in sample weight (g) over each sampling period (h).

### 3.4 Rehydration ratio

As given in Table 3, the final rehydration ratio at the end of the rehydration process was calculated as the weight of the rehydrated sample divided by the weight of the dried sample (*RR1*) increased from 3.88 to 4.51 as rehydration temperature increased from 35 to 60°C. Rajeswari et al. (2013) who used this calculation reported that the rehydration ratio for microwave‐dried (900 W, 180 s) *Amaranthus tricolor* leaves after 3 h 25 min averaged 4.73. Okpala and Ekechi (2014) reported rehydration ratios for sun‐ and shade‐dried West African pepper leaves of 4.26 and 3.83, with blanched leaves having lower rehydration ratios.

**Table 3:** Final rehydration ratios of fresh, dried, and rehydrated amaranth leaves

	Rehy 35°C	Rehy 50°C	Rehy 60°C
RR1 (g/g dry weight)	3.88 ± 0.07^a^	4.34 ± 0.07^b^	4.51 ± 0.06^b^
RR2 (g H_2_O abs/g DM)	3.12 ± 0.07^a^	3.61 ± 0.08^b^	3.80 ± 0.07^b^

RR, Rehydration Ratio. Values are means ± SEM, *n* = 3 per treatment group.

Means in a row without a common superscript letter differ (*p *<* *.05) as analyzed by one‐way ANOVA and the LSD test.

**Figure 3:**
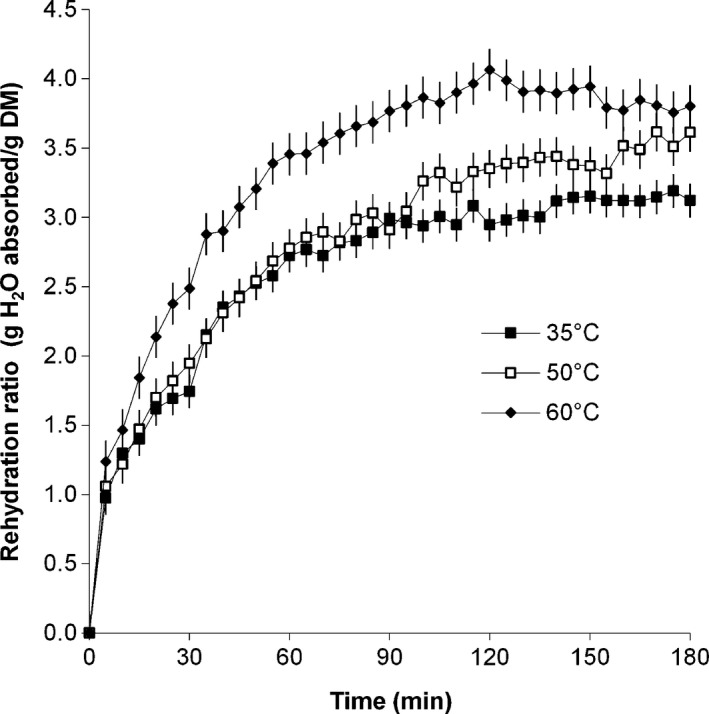
Rehydration ratios of amaranth leaves during rehydration at different temperatures

When calculated as the weight of absorbed water divided by the sample dry matter weight, the rehydration ratio (*RR2*) at the end of the rehydration process increased from 3.12 to 3.18. This rehydration ratio plot as a function of process time is given in Figure 3. Rehydration ratio was significantly affected by time (*p ≤ *.001) and rehydration temperature (*p ≤ *.001).

Rehydration ratio was found to increase rapidly at first, then more gradually until equilibrium was achieved. Rehydration ratio of leaves at all temperatures increased rapidly from zero to 1.0 g H_2_O absorbed/g DM during the first 5 min of the rehydration process, after which, the increase in ratio was more pronounced in leaves rehydrated in water at 60°C. Ratios for leaves rehydrated at 30 and 50°C were similar during 5–100 min of rehydration, but then differences became more noticeable as the ratio for leaves at 50°C increased to a maximum of 3.61, while that for leaves at 30°C peaked at 1.75 g H_2_O absorbed/g DM. Similar curves were reported by Balasubramanian et al. (2011) and Doymaz (2014). Balasubramanian et al. (2011) noted that while higher rehydration temperatures (80°C) resulted in increased rehydration ratios, the color of betel leaves deteriorated at higher temperatures due to leaching. They found that at the lower temperatures of 25 and 40°C, the leaves mostly resembled the fresh leaves, but that overall, a rehydration temperature of 40°C was optimum. Doymaz (2014) found the rehydration curves of mushroom slices at 35 and 50°C to be quite similar for about a third of the rehydration time, with rehydration ratios increasing from zero at the start of the rehydration process to a maximum of between 2 and 3 after 250 min.

### 3.5 Peleg constants

Table 4 gives the Peleg rate constant (*K*_1_) and Peleg capacity constant (*K*_2_) for each rehydration temperature based on plots of *t*/(*M*_0_ *‐M*_e_) versus time (*t*). While a major advantage of the Peleg model is to predict equilibrium moisture content using short‐time experimental data, the range of data selected for the determination of *K*_1_ and *K*_2_ values affects the results obtained (Turhan et al., 2002). Turhan et al. (2002) also noted that while some researchers use absorption data between the beginning of the moisture content curve and somewhere on the curved part in accordance with Peleg (1988), others follow different procedures. This was also found to be the case in this study, and the times corresponding to the curved part of the moisture curve in this study (near or about 115 min depending on the rehydration temperature and specific replicate) were used. Both the Peleg rate constant (*K*_1_) and the Peleg capacity constant (*K*_2_) decreased as rehydration temperature increased, with the temperature effect being significant (*p ≤ *.05) as temperature increased from 50 to 60°C.

**Table 4:** Peleg constants for microwave‐dried amaranth leaves

	Rehydration temperature		
Peleg constants	35°C	50°C	60°C
*K*_1_ (min per g H_2_O/g DM)	6.99 ± 0.424^a^	6.97 ± 0.212^a^	3.72 ± 0.247^b^
*K*_2_ (1/(g H_2_O/g DM)	0.2798 ± 0.0050^a^	0.2696 ± 0.0095^a^	0.2148 ± 0.0036^b^

Values are means ± SEM, *n* = 3 per treatment group.

Means in a row without a common superscript letter differ (*p *<* *.05) as analyzed by one‐way ANOVA and the LSD test.

Equilibrium moisture content values were calculated based on the Peleg constant (*K*_2_) and Equation (8) averaged 3.66, 3.80, and 4.74 g H_2_O/g DM for leaves at 35, 50, and 60°C, respectively. While these values are in reasonable agreement with those obtained experimentally at 35 and 50°C given in Table 2, the *K*_*2*_ value did not adequately predict the *M*_e_ for leaves rehydrated at 60°C. This could be due to increased moisture uptake in these leaves due to additional changes such as cooking.

The Peleg rate constant (*K*_1_) is generally considered to be related to the mass transfer rate and the lower the *K*_1_ value, the higher the initial absorption rate (Turhan et al., 2002). The constant *K*_2_ is inversely related to the absorption ability of foods (Maharaj & Sankat, 2000). The results of hydration studies of various dried materials have supported this. Dadali et al. (2008) reported *K*_1_ values for microwave‐dried (720 W) spinach leaves of 45.5605 s per g H_2_O/g DM rehydrated at 30°C for 300 min. They observed that *K*_1_ values for spinach dried at 360 W decreased from 96.4458 to 33.6346 s per g H_2_O/g DM with an increase in rehydration temperature from 30 to 70°C. Concomitantly, *K*_2_ values were found to decrease from 0.3013 to 0.2677 (1/g H_2_O/g DM). In rehydration studies on basil leaves rehydrated at 30–70°C (Demirhan and Ozbek, 2010), both the kinetic rate constant (*K*_1_) and characteristic constant (*K*_2_) of the Peleg's model are inversely proportional to the rehydration temperature. *K*_1_ values decreased from 139.91 to 97.43 s per g H_2_O/g DM and *K*_2_ values decreased from 0.2498 to 0.2189 (1/g H_2_O/g DM) as rehydration temperature increased from 30 to 70°C. Shafaei et al. (2014) reported a decrease in *K*_1_ value with an increase in temperature for both bean and chickpea, for example, *K*_1_ values for Kabuli chickpea variety decreased from 1.26 to 0.7884 s per g H_2_O/g DM as temperature increased from 5 to 45°C. The increase in Peleg constants with increasing temperature support the result seen earlier, that is, the higher the rehydration temperature, the greater the uptake of water and the greater the equilibrium moisture content. There are also, however, reports of *K*_1_ and *K*_2_ values being temperature independent (Abu‐Ghannam & McKenna, 1997; Okpala & Ekechi, 2014).

## 4 Conclusions

Amaranth leaves were successfully rehydrated in water at 35–60°C. Increasing hydration temperature improved hydration kinetics, however, cooking of leaves occurred at the higher temperatures. With respect to color attributes, the “*L*” values of leaves at 35°C were lower than fresh leaves, indicating that these leaves are darker in color. The results obtained for rehydration data indicate that increase in moisture content and rehydration ratios were more apparent as temperature increased from 50 to 60°C, while rehydration rates were noticeably different during the first few minutes of the rehydration process. Peleg's model can be used to fit the rehydration data with reasonable success and predict equilibrium moisture content values, revealing an inverse relationship between the Peleg rate constant (*K*_1_) and the Peleg capacity constant (*K*_2_) and temperature. As the best compromise between rehydration rate and product quality, a rehydration temperature of 50°C is recommended for microwave‐dried amaranth leaves.

## Funding Information

No funding information provided.

## Conflict of Interest

None declared.
